# A first insight into the genetic diversity of *Mycobacterium tuberculosis *in Dar es Salaam, Tanzania, assessed by spoligotyping

**DOI:** 10.1186/1471-2180-6-76

**Published:** 2006-09-13

**Authors:** Vegard Eldholm, Mecky Matee, Sayoki GM Mfinanga, Manfred Heun, Ulf R Dahle

**Affiliations:** 1 Division of Infectious Disease Control, Norwegian Institute of Public Health, Oslo, Norway; 2 Institute of Nature Resource Management, Norwegian University of Life Sciences, Ås, Norway; 3 Muhimbili Medical Research Centre, Dar es Salaam, Tanzania

## Abstract

**Background:**

Tanzania has a high tuberculosis incidence, and genotyping studies of *Mycobacterium tuberculosis *in the country are necessary in order to improve our understanding of the epidemic. Spoligotyping is a potentially powerful genotyping method due to fast generation of genotyping results, high reproducibility and low operation costs. The recently constructed SpolDB4 database and the model-based program 'Spotclust' can be used to assign isolates to families, subfamilies and variants. The results of a study can thus be analyzed in a global context.

**Results:**

One hundred forty-seven pulmonary isolates from consecutive tuberculosis patients in Dar es Salaam were spoligotyped. SpolDB4 and 'Spotclust' were used to assign isolates to families, subfamilies and variants. The CAS (37%), LAM (22%) and EAI (17%) families were the most abundant. Despite the dominance of these three families, diversity was high due to variation within *M. tuberculosis *families. Of the obtained spoligopatterns, 64% were previously unrecorded.

**Conclusion:**

Spoligotyping is useful to gain an overall understanding of the local TB epidemic. This study demonstrates that the extensive TB epidemic in Dar es Salaam, Tanzania is caused by a few successful *M. tuberculosis *families, dominated by the CAS family. Import of strains was a minor problem.

## Background

In Tanzania, the tuberculosis (TB) incidence doubled between 1990 and 2004 [1]. The rate of all forms of the disease is estimated at 524/100,000 and the rate of new sputum smear positive disease is approximately 157/100,000 [1] with Dar es Salaam contributing about 26% of all TB cases [2]. The World Health Organization estimates that Tanzania has the 14^th ^highest TB burden in the world [1]. Points of concern include the proportion of patients lost to follow-up, currently at 9%, an average diagnostic delay of 6 months, decreasing case detection rate (from 55% in 1997 to 45% in 2004) and the continuing high prevalence of HIV [3]. The high case rate in many African countries has contributed to a rise of the global TB incidence, despite stable or declining rates in the rest of the world [1]. Tanzania with its 37 million inhabitants, has 701 district laboratories diagnosing TB, three laboratories culturing *M. tuberculosis *and one National reference laboratory that perform drug susceptibility testing of *M. tuberculosis *isolates. Measures are undertaken to establish molecular genotyping methods such as spoligotyping [4], but currently no laboratory in Tanzania offers this service. Previous studies have described the molecular epidemiology of Tanzanian *M. tuberculosis *collections from the first half of the 1990s [5-7]. Spoligotyping is a PCR-based fingerprinting method that detects the presence or absence of 43 defined spacers situated between short direct repeat (DR) sequences in the genomes of members of the *M. tuberculosis *complex. Important advantages of spoligotyping are that it is cheap, easy to perform and fast. In addition, it has been demonstrated that the results are highly reproducible [8]. Unique to spoligotyping results are tools like the SpolDB4 database [9] and the web-based computer algorithm 'Spotclust' [10] that can be used to assign new isolates to families, subfamilies and variants (SpolDB4 only). SpolDB4 is the largest and most up to date available global database for spoligotypes. For previously not reported spoligopatterns, the 'Spotclust' database is a good additional tool in that it can assign these patterns to families by using a computer algorithm based on studies of SpolDB3 [10]. The results from local studies can thus be analyzed and compared to the global *M. tuberculosis *population. This may help us better understand the world-wide spread of common *M tuberculosis *families and subfamilies. In order to improve our understanding of the TB epidemic in this high-incidence country, the current ongoing study included *M. tuberculosis *strains collected in Dar es Salaam during October and November 2005. We describe the diversity of *M. tuberculosis *isolates from Dar es Salaam, Tanzania, based on spoligotyping, and identify the families and subfamilies responsible for the current persistence and spread of TB in this high-incidence community.

## Results

### Genetic diversity and family assignment

The 147 analyzed isolates gave 76 different spoligopatterns resulting in an overall diversity of 52%: 57 spoligopatterns occurred only once and 19 patterns comprised 90 of the isolates (61%) (table 1). Forty-nine (64%) patterns had not been described previously. The SpolDB4 database assigns isolates to families, subfamilies and often to variants, whereas 'Spotclust' assigns isolates to families and subfamilies, but is not designed to assign isolates to variants. Four spoligopatterns were assigned to different families and nine patterns were assigned to different subfamilies by the two methods. SpolDB4 assigned names were used whenever a spoligopatterns was found in the database, as this database is much larger than the SpolDB3 database, on which the 'Spotclust' algorithm is built. Patterns not found in SpolDB4 were assigned to families and subfamilies by 'Spotclust'. The family assignment showed that 37% of the isolates belonged to the Central Asian (CAS) family, 22% to the Latin American Mediterranean (LAM) family, and 17% to the East-African Indian (EAI) family. These three main families thus accounted for 76% of the incidences in Dar es Salaam. This family assignment also includes the spoligopatterns not described before. Eight isolates lacked spacers 4–7, 10 and 20–35, typical of the CAS1-kili variant, but in addition, they all also lacked spacer 2 (table 2). This spacer is typically present in CAS1-kili lineages and its absence has not previously been reported in these variants. We propose to name these variants CAS1-DAR, since they appear to be abundant in Dar es Salaam.

The rate of diversity (number of spoligotypes divided by the number of isolates) within each main family varied substantially and was 27, 54 and 72% for CAS, LAM and EAI, respectively. This may indicate that the CAS family is best adapted to spread within this community. The diversity of the *M. tuberculosis *population in Dar es Salaam (52%) was comparable to that described in previous studies from Tanzania [5-7]. In Delhi, India the genetic diversity of the *M. tuberculosis *population is 42% [11], but it is only 25% in Harare, Zimbabwe [12]. Thus, the diversity in high-incidence countries varies greatly and may be difficult to estimate without molecular epidemiological studies.

### Phylogenetic studies

**Figure 1:**
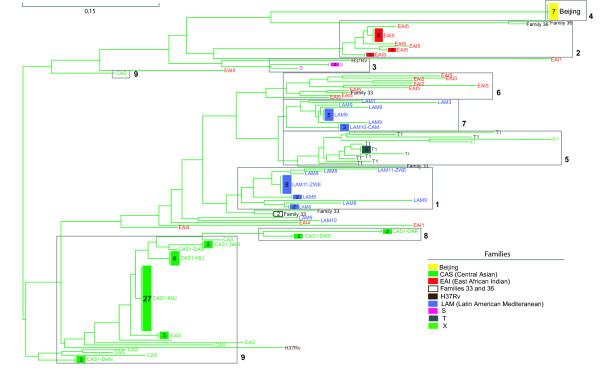
**Neighbor-joining tree of the 147 isolates of *M. tuberculosis***. Neighbor-joining tree of the 147 isolates of *M. tuberculosis*. The isolates are colour-coded according to family assignment. The nine groups identified by Structure are identified by grey open boxes. One CAS isolate (*) assigned to the large CAS group is shown in a separate box. Only isolates showing > 65% membership in a group are included in the boxes. For convenience, the NJ tree is rooted by mid-point rooting.

A Neighbor-joining (NJ) tree of all the isolates is shown in figure 1. The main families were well distinguished and a high diversity within and between families were observed. To confirm the reliability of the NJ tree, the program 'Structure' was applied on the underlying 43-digit binary spacer codes. The open boxes in figure 1 demonstrate the nine groups found to be the most likely number; the NJ branches were supported by the grouping via 'Structure'.

## Discussion

The current study demonstrated that most isolates had at least one other closely related isolate in Dar es Salaam. Based on these preliminary findings, the TB epidemic appeared to result from a gradually evolving *M. tuberculosis *population rather than imported strains. A spoligotyping study conducted in the Ouest province of Cameroon found that 193 of 413 *M. tuberculosis *isolates belong to the Cameroon family (LAM10-CAM) [13]. In Harare, Zimbabwe, 68 of 214 isolates are LAM11-ZWE variants [12]. Of the 147 isolates in this study, three and eight isolates belonged to these variants respectively. The scarcity of these strains, abundant in other African countries, also indicated that the TB epidemic in Dar es Salaam is local and well established.

When live cultures are not available, two PCR based methods are preferred in order to determine the degree of clustering among *M. tuberculosis*. Such complementary studies will be undertaken for the current population but are not included in the current paper.

Spoligotyping is not necessarily the best method for phylogenetic studies, since it targets a small region of the genome. The knowledge of the evolution of this region is limited. It has however been proposed that transposition of insertion sequences can lead to convergence of spoligopatterns and that the evolution of the region is unidirectional (spacers can be lost but not gained). Also, contiguous blocks of spacers and DRs can be lost in single events [14]. These facts may obscure phylogenetic analyses using simple distance based methods. Despite these weaknesses, spoligotypes have been shown to correlate quite well with single nucleotide polymorphisms (SNP), with the T family, constituting only 10 isolates in this study, as a notable exception. For these reasons a NJ-tree was used to illustrate the current results.

The success of the CAS family in particular, but also the LAM and EAI families in this community is intriguing. The low diversity of the highly prevalent CAS family in this study may indicate that the family is spreading rapidly, but could also reflect a slower evolution of the DR region which could possibly be a result of the missing spacers in the central part of the spoligopatterns of these strains.

The success of these three families suggests a possible co-evolution between specific *M. tuberculosis *families and host population, the molecular basis of which remains to be elucidated. A study conducted in San Francisco supports the idea of co-evolution between this pathogen and host populations [15]. In order to document such possible co-evolution, large populations should be preferred. Internationally standardized methods such as spoligotyping and MIRU-typing, as well as SNP and deligotyping, enable comparison of *M. tuberculosis *genotypes between studies conducted at different times and locations. This facilitates inter-study comparison and helps generate large populations for such evolutionary scenarios. It should be noted that the current study represents a short time period and a small collection of strains. This complicates interpretation of recent transmission and hampers comparisons of genetic diversity with that found in studies conducted over a longer period of time. The use of different genotyping methods also makes direct comparison with previous studies in Tanzania [5-7] difficult.

Recent findings suggest that the tubercle bacillus emerged in Africa and may have spread globally in parallel with the human migrations out of Africa [15,16]. Another study have however identified India as the center for the evolutionary radiation of *M. tuberculosis *[17]. These theories are not mutually exclusive; as the spread to India might represent an early and evolutionary important step in the radiation of *M. tuberculosis *out of Africa. The CAS- and EAI-families which this study found to be abundant in Dar es Salaam, have previously been identified to have the most ancestral roots [17]. We demonstrate that the Beijing family, which is highly prevalent in many Asian locations, is not common in the current population. It therefore appears unlikely that import of strains from Asia have had a major impact on the *M. tuberculosis *population in Dar es Salaam. The sensitivity of spoligotyping alone is insufficient for pinpointing evolutionary origins and direction of movement, but the current findings lend support to a view of an early African origin of *M. tuberculosis*.

Spoligotyping is inexpensive, fast, simple and reliable. By using this method one can identify outbreaks, support community-based contact tracing, describe the diversity of a *M. tuberculosis *population, and compare this population to that in other parts of the world. Implementation of spoligotyping as a routine method for molecular epidemiological studies of *M. tuberculosis *isolates, appear to represent a valuable investment in many high-incidence countries.

## Conclusion

Spoligotyping is very useful to gain an overall understanding of the local TB epidemic. This study demonstrated that the extensive TB epidemic in Dar es Salaam, Tanzania was caused by a few successful *M. tuberculosis *families, dominated by the CAS family. Import of new strains was a minor problem.

## Methods

### DNA extraction and spoligotyping

Isolates of *M. tuberculosis *were collected from sputum smear positive TB cases in consecutive patients in Dar es Salaam during October and November 2005. Heat-killed samples were shipped to Norway, DNA was extracted [18] and a total of 147 *M. tuberculosis *isolates were spoligotyped according to Kamerbeek et al. [4].

### Family assignment

The obtained spoligopatterns were first compared to the SpolDB4 database [9] and assigned to families and subfamilies. Second, in order to assign names to the isolates not found in the SpolDB4 database, the spoligopatterns were analyzed with 'Spotclust'[10], using a mixture model built on the SpolDB3 database. This model takes into account knowledge of the evolution of the DR region and assigns spoligopatterns to families and subfamilies.

### Phylogenetic analyses

A NJ-tree [19] was constructed by converting the presence or absence of 43 defined spacers of the 147 isolates into a Jaccard [20] based pair-wise distance matrix with the computer program 'NTSYSpc' (Exeter Software Co., New York). Without conversion to distance, to verify the NJ tree, the spacer data were directly used by the program 'Structure' [21] to identify groups into which the individual isolates fit best and to calculate the best number of groups explaining the whole data set (run with a no-admixture-model, and a burn-in of 100000 repeats and 400000 Markov Chain Monte Carlo repeats, 65% assigned membership to a group was used as a threshold value in figure 1).

## Competing interests

The author(s) declare that they have no competing interests.

## Authors' contributions

VE carried out the DNA extraction, genotyping, data analyses and participated in the design of the study. MM conceived the study and collected, cultured and identified the bacterial isolates. SGMM participated in the design of the study and collected, cultured and identified the bacterial isolates. MH participated in the data analyses and in the design of the study. URD conceived the study, supervised the DNA extraction, genotyping and data analyses. All authors contributed in the writing of the article, read and approved the final manuscript.

## Figures and Tables

**Table 1:** Spoligopatterns and family assignment

**Spoligotype**	**Shared type**	**SpolDB4 ID**	**SpotClust ID**	**SpotClust probability**	**No. of isolates with identical pattern**
	1	Beijing		1	7
	4	LAM3/S		1	1
	1468	LAM11-ZWE		0,99	1
	NEW		LAM8	0,99	1
	964	LAM9		0,97	1
	NEW		LAM9	0,99	1
	NEW		LAM9	0,99	1
	NEW		LAM3	0,99	1
	NEW		LAM8	0,99	1
	NEW		LAM10	0,99	1
	NEW		LAM8	0,99	1
	NEW		LAM8	0,99	2
	150	LAM9		0,99	1
	811	LAM4		0,99	2
	59	LAM11-ZWE		0,99	8
	NEW		LAM9	0,99	1
	1530	LAM9		0,99	1
	42	LAM9		0,99	5
	61	LAM10-CAM		0,99	3
	288	CAS2		1	1
	NEW		CAS	0,99	1
	NEW		CAS	1	1
	1675	CAS1-kili		1	6
	NEW		CAS	1	1
	21	CAS1-kili		1	27
	NEW		CAS	1	1
	22	CAS		1	3
	486	CAS		1	1
	NEW		CAS	1	1
	NEW	CAS1-DAR		1	2
	NEW	CAS1-DAR		1	2
	NEW	CAS1-DAR		1	3
	NEW	CAS1-DAR		1	1
	NEW	CAS1-DAR		1	1
	NEW		EAI4	0,96	1
	NEW		EAI5	0,99	1
	733	EAI5		0,99	1
			EAI5	1	1
	NEW		EAI5	0,99	1
	NEW		EAI2	0,99	1
	NEW		EAI1	0,99	1
	1864	EAI5		1	1
	NEW		EAI5	1	2
	NEW		EAI5	1	2
	NEW		EAI5	1	1
	8	EAI5/EAI3		1	6
	NEW		EAI2	0,99	1
	NEW		EAI3	0,99	1
	NEW		EAI5	0,99	1
	NEW		EAI5	0,99	1
	NEW		EAI4	0,99	1
	129	EAI5		0,99	1
	53	T1		0,99	3
	NEW		T1	0,99	1
	NEW		T1	0,99	1
	420	T2		0,91	1
	NEW		T1	0,99	1
	205	T1		0,99	1
	NEW		T1	0,99	1
	NEW		T1	0,99	1
	1166	T1		0,99	1
	NEW		T1	0,99	1
	NEW		Family36	1	1
	NEW		Family33	1	1
	NEW		Family33	0,99	1
	NEW		Family33	1	1
	NEW		Family33	0,99	2
	NEW		H37Rv	0,98	1
	NEW		H37Rv	0,99	1
	NEW		S	0,98	2
	NEW		S	0,98	1
	NEW		X1	0,91	1
	402	U		0,85	1
	354	U		0,99	3
	1196	U		0,99	1

**Table 2:** The CAS1-DAR variants. Four previously unreported variants of the CAS1 subfamily. The variants are collectively named CAS1-dar in this study.

**Spoligopattern**	**Octal code**	**No of isolates**
	503367400001401	2
	503367400001471	2
	503367400001771	3
	503377400001771	1
